# Manufacturing carbon storage sintered body using microwave-selective and high-speed heating techniques

**DOI:** 10.1038/s41598-023-32136-z

**Published:** 2023-03-29

**Authors:** K. Kashimura, A. Oshita, T. Miyata, S. Segawa, H. Yokawa, K. Tendo, K. Kurooka

**Affiliations:** 1 Faculty of Engineering, Chubu University, 1200 Matsumoto-cho, Kasugai, Aichi 487-8501 Japan; 2 Chugoku Kouatsu Concrete Industries Co., Ltd., 4-33 Komachi, Naka-ku, Hiroshima 730-0041 Japan

**Keywords:** Carbon capture and storage, Fossil fuels, Civil engineering

## Abstract

Microwave sintering of fly ash samples with large amounts of unburned carbon and CaCO_3_ was examined in this study. To this end, CaCO_3_ was mixed with fly ash sintered body to fix CO_2_. The decomposition of CaCO_3_ was observed when the raw material was heated to 1000 °C using microwave irradiation; however, a sintered body containing aragonite was obtained when the raw material was heated to 1000 °C with added water. Further, carbides in the fly ash could be selectively heated by controlling the microwave irradiation. The microwave magnetic field created a temperature gradient of 100 °C in a narrow region of 2.7 μm or less in the sintered body, and it helped suppress the CaCO_3_ decomposition in the mixture during sintering. By storing water in the gas phase before spreading, CaCO_3_, which is difficult to sinter using conventional heating, can be sintered without decomposing.

## Introduction

Decarbonization is gaining considerable interest for the realization of a sustainable society. In November 2021, the 26th Conference of the Parties to the United Nations Framework Convention on Climate Change was held in the United Kingdom, wherein Japan's ambitious efforts in the field of climate change were disseminated^1^. The swift transition to a carbon–neutral society by 2050 is required not only in Japan but also worldwide. In conjunction, increasing attention is devoted toward the effective utilization of industrial waste, such as fly ash discharged from coal-fired power plants and concrete slag. Attempts to capture and fix CO_2_ in concrete and slag have been successful^2–11^. Cement production produces approximately 0.8 kg of CO_2_ per kg of cement, which amounts to approximately 5–8% of global CO_2_ emissions^2^. Several studies on fixing CO_2_ to concrete have focused on the carbonation of concrete slurry waste (CSW). Research on thermodynamically carbonizing CSW in a controlled environment has been reported in basic research^2–4^, applied research^4,5^, theory^6^, and scale-up^7^, and it has been gaining increasing attention as a carbon capture and storage technology that can permanently isolate CO_2_. Further, the carbonation of slag has gained interest as a CO_2_ storage technology in the steel field. Slag is a by-product of iron production. The global iron slag production in 2021 was estimated to be between 340 and 410 million tons^8^, and research on precipitating CaCO_3_ by chemically converting these using alkaline compounds has attracted considerable attention^9–11^. These studies are characterized by the ability to obtain high-purity CaCO_3_ in a powder form. However, there is a need for a technique to make the obtained powder-shaped CaCO_3_ into a structural material to permanently fix CO_2_.

We focus on microwave heating as a technology to sinter materials without decomposing CaCO_3_ powders. Many researchers have investigated microwave heating in the field of ceramic sintering because it allows the rapid heating of objects^12–15^. In addition, research in the last few years has confirmed that microwaves create a temperature gradient of several hundred degrees in a narrow region ranging from 4.7 to 60 nm of the mixture^16–18^. Thus, materials other than CaCO_3_ powder can be selectively heated by employing these two characteristics well, and the mixture can be sintered before CaCO_3_ decomposes.

We aim to create a fly ash sintered body that contains CaCO_3_ by utilizing the characteristics of microwave heating. Fly ash is a by-product of thermal power plants, which produce approximately 500 million tons of it annually^19–22^. It is costly to dispose of fly ash because it contains approximately 10–20% unburned carbon^20,23^. A CO_2_ reduction effect can be achieved if a sintered body can be formed with carbon trapped in fly ash. Thus, a double carbon storage effect can be realized if a heating method that does not significantly burn the carbon content of fly ash and decompose CaCO_3_ is developed.

To this end, we exploit the characteristics of microwave heating to create a sintered body containing CaCO_3_ in a single operation. Microwaves can heat fly ash to 1000 °C within tens of seconds^24^, which indicates that sintering can be completed before the water molecules essential for CaCO_3_ formation diffuse. In addition, we investigate an irradiation method that suppresses the decomposition of CaCO_3_ and the combustion of unburned carbon by precisely separating the electric and magnetic fields of microwaves and irradiating the fly ash raw material. The temperature of fly ash was measured at a spatial resolution on the order of micrometers, and the relationship between the microwave irradiation method and narrow temperature distribution was investigated.

## Methods

The microwave absorption of fly ash is highly dependent on carbon concentration; however, the amount of carbon remaining in fly ash varies significantly from one power station to another. This difference in the amount of unburned carbon arises because of the difference in fossil fuels used as raw materials for power generation. Therefore, we obtained fly ash samples from two actual power plants (denoted as S and M). As expected, the S- and M-fly ash samples had different carbon contents (Table 1). The two types of fly ash were mixed with CaCO_3_ to produce the samples used in this study. Further, NaCl was employed as a sintering aid.

**Table 1:** Chemical composition of fly ash and garnet samples determined by XRF.

Sample	Composition (mass%)							
	C	O	Al	Si	Fe	Na	Cl	Other
Incineration ash (S-fly ash)	7.22	50.72	8.54	26.07	2.28	0.42	–	4.75
Carbon-containing ash (M-fly ash)	23.63	48.27	7.90	14.59	1.75	0.24	–	3.62
Mixed sample	10.37	47.16	9.28	19.86	1.69	0.64	0.62	6.26

S-fly ash, M-fly ash, NaCl (97.7 mass%, Arashio Co. Ltd., Shizuoka), and CaCO_3_ (99.5 mass%, Junsei Co. Ltd., Tokyo) were mixed to prepare a sintered raw material (S-fly ash:M-fly ash:NaCl:CaCO_3_ = 4:1:1:1 [mass ratio]). This composition was determined using the SiO_2_-Al_2_O_3_-Na_2_O phase diagram, instead of the amount of unburned carbon^25^. The state diagram shows that the melting point of fly ash is approximately 1060–1160 °C when the SiO_2_:Al_2_O_3_:Na_2_O mass ratio is approximately 5:2:1. Therefore, it was considered that the temperature required for sintering could be lowered.

The mixture (weight: 1.5 g; standard deviation: 0.001 g) was loaded into a quartz holder, which was tapped 100 times to suppress any changes in volume. Then, the quartz holder was loaded into a cavity resonator, wherein the sample to which a small amount of pure water (0.8 g) was added was also heated to recover CaCO_3_ decomposed by heating.

**Figure 1:**
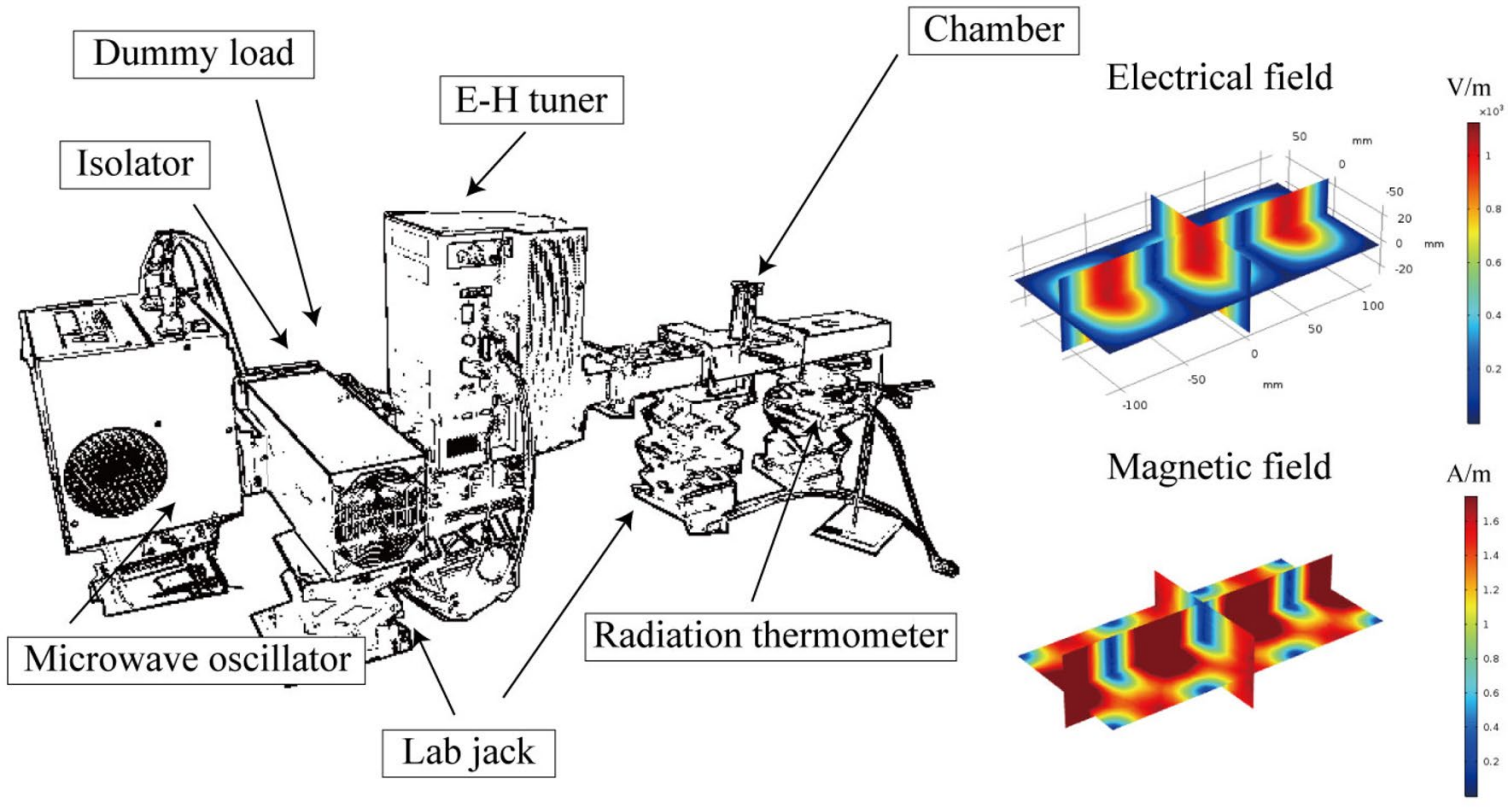
Schematic illustration of the single-mode heating device and the calculated electromagnetic field intensity distribution inside the device during microwave irradiation. The microwave power is assumed to be 1 W.

We employed separated microwave fields with a frequency of 2.45 GHz as the heating method. The system had six waveguides (109.1 × 56.4 × 149.3 ± 5 mm) combined with a magnetron oscillator, E–H tuner, plunger, and dummy load as shown in Fig. 1. The microwaves were focused using a slit and they formed a TE103 wave within the cavity. The slit had a 52-mm slit parallel to the orientation of the electric field. The plunger was placed at the end of the waveguide. This system enabled us to spatially separate the electric and magnetic fields of the microwaves^26,27^. The sample was placed at an electric field node (denoted by *E*_max_, where the magnetic field is zero) or magnetic field node (denoted by *H*_max_, where the electrical field is zero). When the sample was heated by microwave, 0.4 L/min CO_2_ gas was induced into the cavity. The temperature of the reactants was monitored using a radiation thermometer (FTZ6-R220-5S22, Japan Sensor Corp.) The electrical permittivity was investigated at various temperatures using a cavity perturbation method to understand the heating behavior^28,29^. The gas emitted while heating the fly ash was analyzed using Q-mass (PrismaPro QMG250, Hakuto Co. Ltd., Tokyo).

After heating, the volume reduction ratio, residual carbon, and strength of the fly ash samples, which play an important role in the utilization of fly ash, were investigated. The volume reduction ratio and amount of residual carbon were measured using Archimedes’ method and X-ray fluorescence (XRF), respectively. Finally, mono-axial compression tests were conducted to measure the strength of the samples.

The Thermera-NIR2 system (Mitsui Photonics Ltd.) was employed to measure the temperature distribution obtained from two wavelengths (800 and 975 nm)^30,31^, which resulted in measuring the area temperature at each pixel of the image. Using this system, we obtained the temperature with 2.7 μm/pixel over 800 °C. The accuracy of the analyzed temperature was ± 1%.

## Results and discussion

**Figure 2:**
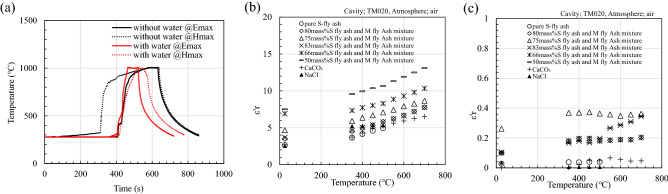
(**a**) Temperature change when the 1.5 g sample with (or without water) is heated by a microwave electric field (or magnetic) field (holding time: 1 min, holding temperature: 1000 °C, 0.4 L/min CO_2_). Temperature dependence of the (**b**) real and (**c**) imaginary dielectric constants of the fly ash raw material measured by the resonant perturbation method (TM020, air).

Microwaves quickly heat fly ash. Figure 2a shows the temperature change when 1.5 g of the mixture material is heated by a microwave electromagnetic field. The fly ash shows good microwave absorption regardless of the microwave irradiation conditions; however, electric field heating shows a higher gradient than that of magnetic field heating near 1000 °C. This phenomenon is attributed to the improvement of the dielectric constants of the components of the mixture at high temperatures. Figure 2b,c show the temperature dependence of the real and imaginary dielectric constants of the components of the mixture measured using the resonant perturbation method, respectively. Here, it is difficult to measure the dielectric constant using the coaxial transmission and resonance perturbation methods because M-fly ash has a large dielectric loss. Thus, the composition of M-fly ash is increased, and the microwave absorption dependence is measured. Figure 2c shows that increasing the amount of M-fly ash improves microwave absorption, and CaCO_3_ and NaCl barely absorb microwaves; this indicates that the unburned carbon remaining in M fly ash significantly contributes to microwave absorption.

**Figure 3:**
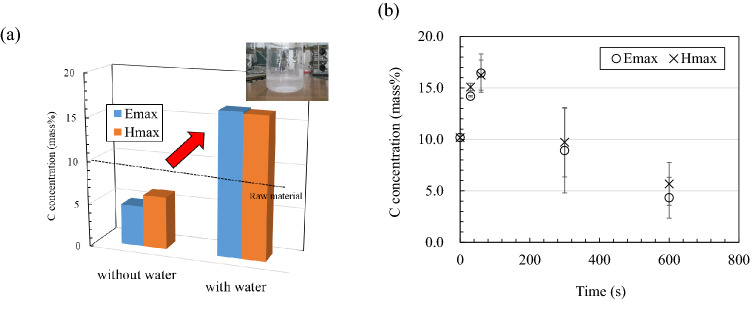
(**a**) Comparison of carbon concentration in the mixture with or without added water after heating with microwave electric field and magnetic field and (**b**) the sintering time dependence of carbon concentration in the sintered body.

Figure 3a shows the carbon content of the mixture sintered at 1000 °C. The mixture before microwave heating contains 10.2 mass% carbon; this is from both unburned carbon and the carbon derived from CaCO_3_. The mixture without water was heated to 1000 °C with microwave irradiation (1 min), following which a decrease in carbon concentration was confirmed. This decrease in the carbon concentration can be attributed to the decomposition of CaCO_3_ and the combustion of carbon. An increase in the carbon concentration can be confirmed in the mixture to which water is added, after the same amount of heating. A gas that makes the lime water cloudy can be obtained when the sintered mixture to which water is added is pulverized again and heated at 1000 °C in an N_2_ atmosphere, as shown in Fig. 3a. This is attributed to the burned carbon causing elements other than carbon to escape into the gas and suppress decomposition and combustion of CaCO_3_ and carbon, respectively. Figure 3b shows the heating time dependence of the carbon content of the sintered body obtained by heating the mixture with added water at 1000 °C. Both electric and field heating show high carbon content when the retention time is short; this is because CaCO_3_ decomposition and carbon combustion do not progress sufficiently owing to the short sintering time. Because silicon, calcium, oxygen, and other elements scattered from the mixed fly ash, the carbon concentration increased (Table S1). The carbon concentration decreases with an increase in the heating retention time. This decrease in carbon concentration in the sintered body of the mixture is smaller in magnetic field heating than that in electric field heating.

To improve the accuracy of the measurement, the sintered body after microwave treatment was pulverized and reheated to 1000 °C. in a nitrogen atmosphere. The obtained flue gas was passed through a Ca(OH)_2_ solution, and a cloudy state was observed (bubbling test). This test result supports the hypothesis that the sintered body is heated in a magnetic field and the shorter the burning time, the more CO_2_ is contained (Fig. S1).

Furthermore, thermogravimetry differential thermal analysis (TG–DTA) was used to detect CaCO_3_. The raw material was heated at 1000 °C for 30 s by microwave electric or magnetic field heating, and the obtained sintered body was analyzed by TG–DTA. According to the TGA results (Fig. S2), the sintered body heated by the microwave electric field showed two weight losses of − 1.19 wt% (*N* = 3, 400–600 °C) and − 2.97 wt% (*N* = 3, 600–900 °C). On the other hand, the sintered body heated by the microwave magnetic field showed wight losses of − 1.94 wt% (*N* = 3, 400–600 °C) and − 4.78 wt% (*N* = 3, 600–900 °C). According to the DTA results, both sintered bodies showed endotherms in the ranges of 400–600 and 600–900 °C. CaCO_3_ decomposes at 400–900 °C, which is an endothermic reaction. Therefore, both sintered bodies contain CaCO_3_ and magnetic field heating produce sintered bodies containing more CaCO_3_ than sintered bodies produced by electric field heating.

**Figure 4:**
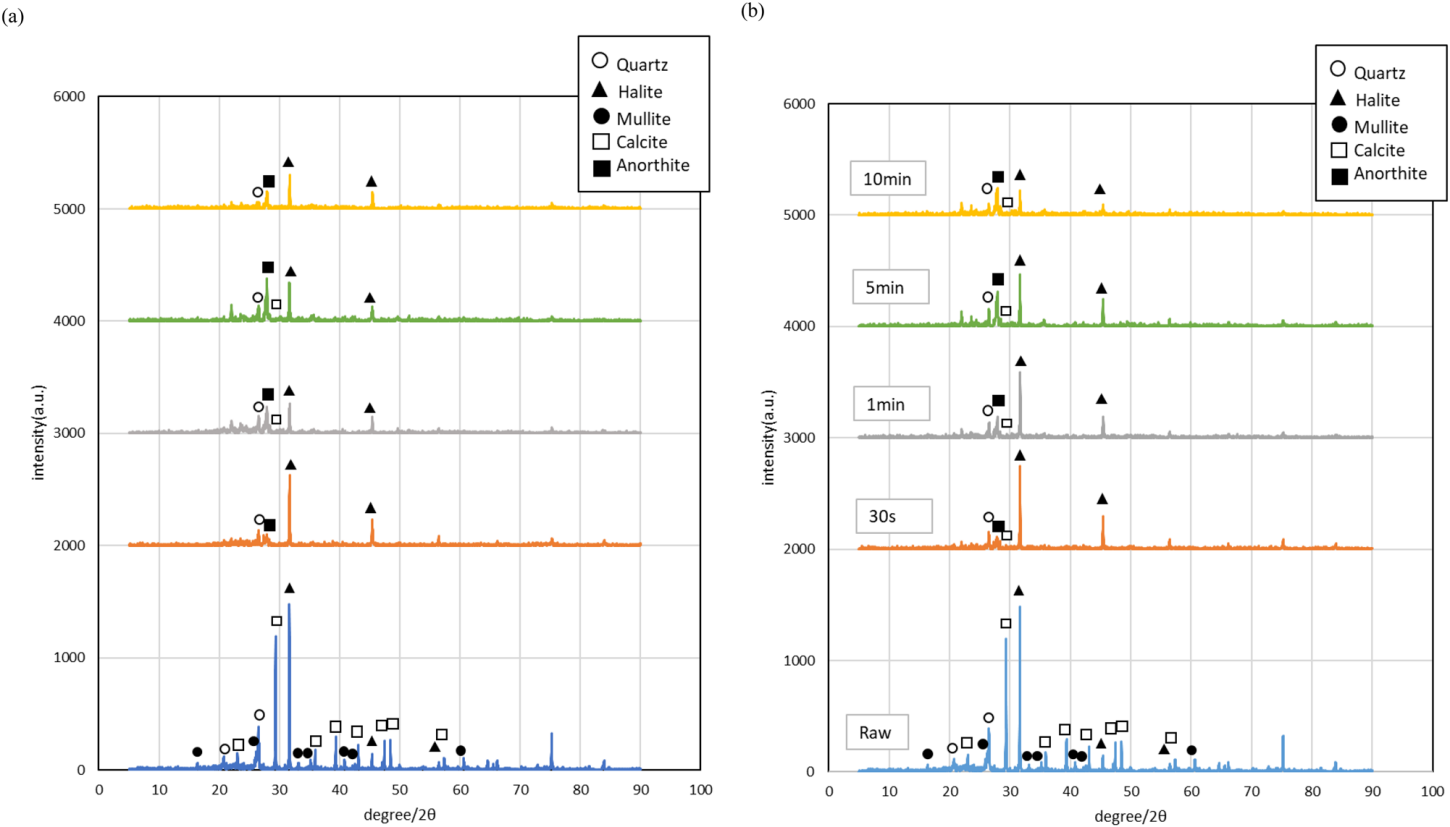
XRD patterns of the water-added mixture (**a**) after microwave electric field heating and (**b**) after microwave magnetic field heating.

Figure 4a shows the XRD patterns of the sintered body obtained by heating the mixture with water using a microwave electric field. The measurement result shows the peak of aragonite, which is a crystal of CaCO_3_. Microwave-heated sintered bodies showed a crystal structure with anorthite as the main component. Also, the sintered body heated by the microwave magnetic field showed a calcite peak. This peak disappears as the heating time increases as shown in Fig. 4b. Considering this result together with the results of TG–DTA and chemical analysis, it can be confirmed that in the microwave heating magnetic field, the amount of calcite observable by XRD remains without being decomposed.

**Figure 5:**
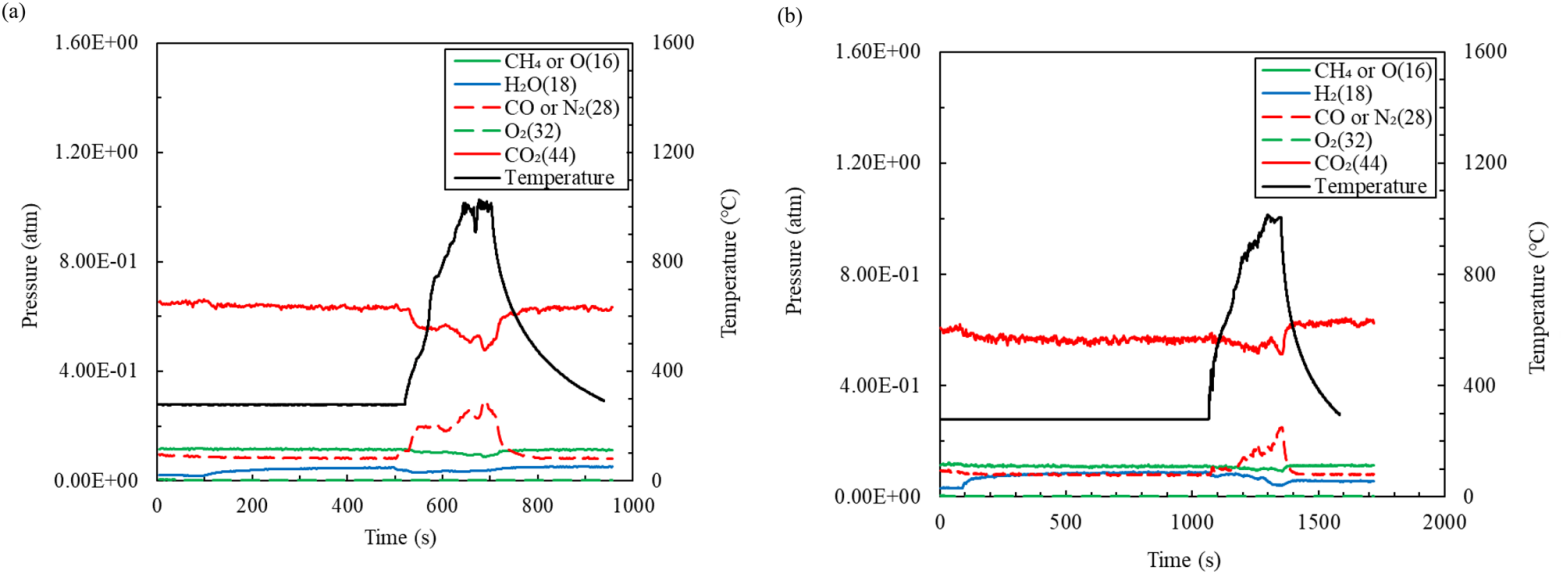
Temperature change and exhaust gas analysis results as a function of time when the mixture is heated with (**a**) a microwave electrical field and (**b**) a magnetic field.

The microwave magnetic field suppresses the decomposition of carbon compared with that in the microwave electric field when the mixture is sintered. This tendency was confirmed from the results of the exhaust gas analysis. Figure 5a shows the temperature change and exhaust gas analysis results versus time when the mixture is heated by a microwave electric field. The exhaust gas analysis was conducted by operating an exhaust gas by Q-mass; the vertical axis is partial pressure calculated from the ion current and the carrier gas is CO_2_ (0.4 L/min). The CO_2_ partial pressure decreased to 65% because the pressure inside the furnace is negative because the exhaust gas is pulled by a vacuum pump when the carrier gas is introduced. As shown in Fig. 5a, an increase in the gas with a mass number of 28, which corresponds to CO, can be confirmed immediately after the start of microwave irradiation. Therefore, it is inferred that carbon combustion occurs because CO gas is only generated when carbon is burned at a high temperature, according to thermodynamics principles^32^. Figure 5b shows the temperature change over time and the flue gas analysis results when the mixture is heated in a microwave magnetic field. The gas with mass number 28, corresponding to CO, increases when the mixture reaches 1000 °C. Furthermore, the CO gas returns to the baseline as soon as the mixture temperature decreases, and only a small amount of CO gas was released compared to the electric field heating. Therefore, for in situ microwave heating, the reaction in which carbon burns and is converted into CO gas is suppressed. Considering that carbon is well heated in microwave electric and magnetic fields, we concluded that CaCO_3_ around carbon is stable and cannot react in a microwave electric field due to its low temperature.

**Figure 6:**
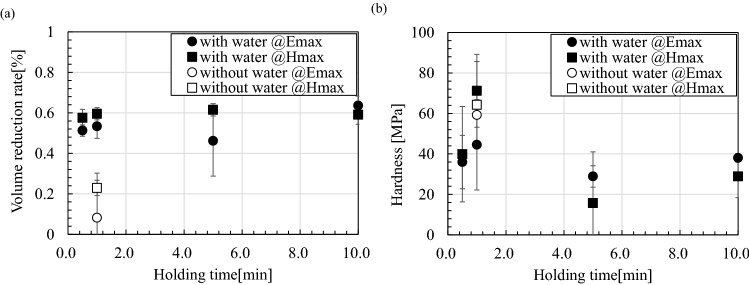
Retention time dependence of the (**a**) volume reduction rate and (**b**) rigidity of the mixture sintered at 1000 °C.

Volume reduction rate and rigidity are important parameters that determine the engineering utility value of the sintered body. Therefore, the effect of adding water on the volume reduction rate and rigidity of the fly ash sintered body is investigated. Figure 6a shows the retention time dependence of the volume reduction rate of the mixture sintered at 1000 °C. Here, the error bar adopts the standard deviation measured three times. Figure 6a confirms that the addition of water is effective for improving the volume reduction rate. In addition, magnetic field heating is more effective in reducing the volume of the mixture than electric field heating when the holding time is short. This implies that the addition of water and magnetic field heating reduce air bubbles in the sintered body. Figure 6b shows the retention time dependence of the rigidity of the mixture sintered at 1000 °C; the addition of water does not affect the rigidity of the sintered body. Further, the magnetic field heating is more effective for the rigidity of the mixture than electric field heating when the holding time is short; electric field heating is more effective than magnetic field heating when the holding time is long.

However, the question “Why does the addition of water suppress the decomposition of CaCO_3_?” remains unanswered. This phenomenon can be explained by considering that the high-speed heating property of microwaves completes sintering before water diffuses. Once the temperature has risen, the sintered body undergoes the process of decreasing temperature. The presence of water and CaO in the thermodynamically stable region of CaCO_3_ promotes the carbonation of the sintered body. This hypothesis explains that the addition of water and shortening of sintering time are effective for carbon storage. However, the exhaust gas analysis results indicate that the carbon combustion temperature drops when heated by a microwave magnetic field, and therefore, it is necessary to consider the cause.

It is necessary to discuss mesoscale thermodynamics to consider the current microwave chemistry. Thus far, several researchers have reported that microwave-heated materials behave differently than materials heated via conventional methods^33–36^. In recent years, research has clarified that the inhomogeneity of the material creates a mesoscale superheat point on the object to be heated^16–18^; further, a superheat point is observed in this system.

**Figure 7:**
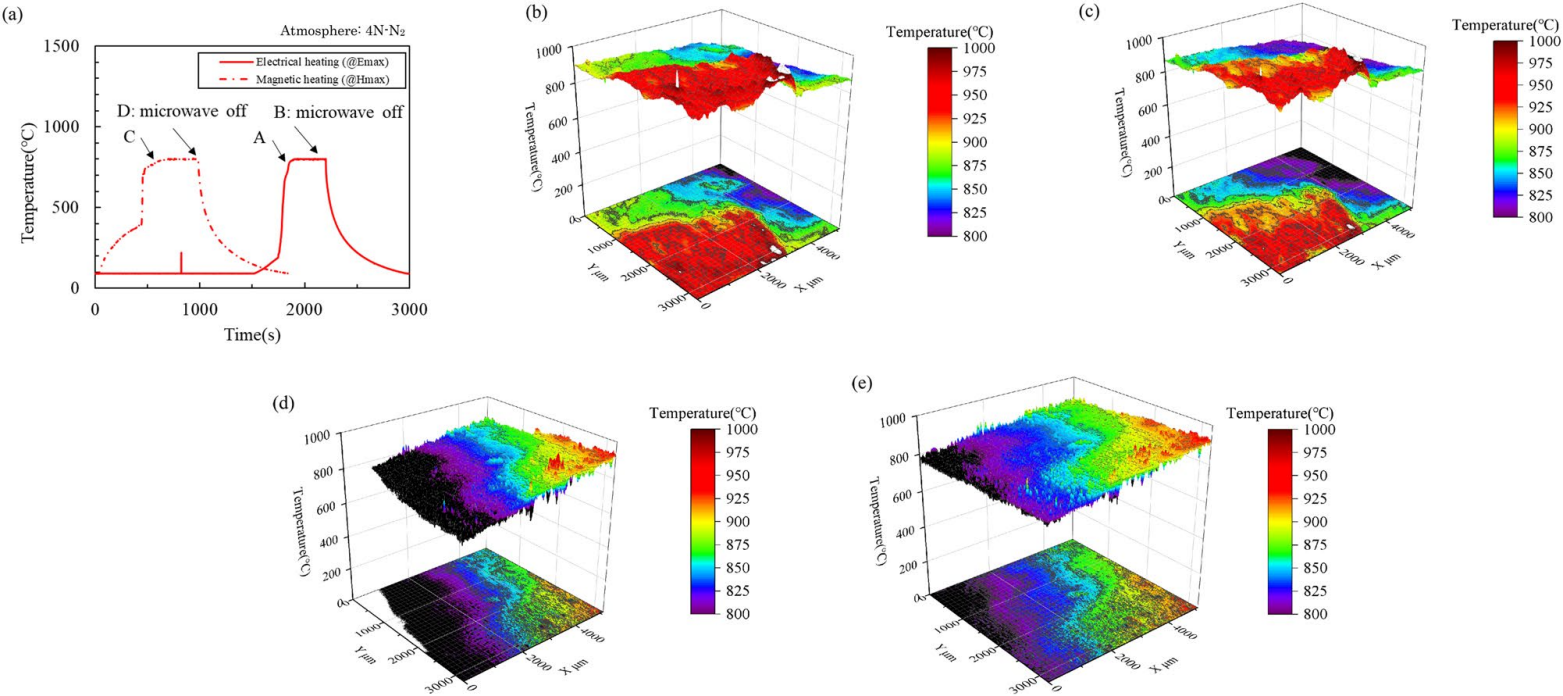
(**a**) Temperature change in the mixture with time and (**b**–**e**) the narrow temperature distribution at points A and D in the microwave electric field and magnetic field heating.

Figure 7a shows the temperature change of the mixture with time and (b–e) show the measurement result of the narrow temperature distribution at points A and D in the microwave electric field and magnetic field heating; each pixel in Fig. 7b–e is 2.7 μm. Figure 7b,c show that the mixture heated by the microwave electric field has a superheat point the size of several hundred micrometers, and this superheat point is approximately 20–50 °C higher than the surroundings and maintained for 5 min. The mixture heated by the microwave magnetic field has a superheat point the size of several micrometers or less, and it is approximately 50–100 °C higher than the surroundings and is maintained for 5 min (Fig. 7d,e). In the monochromatic thermometer measurement results shown in Fig. 7a, the microwave electric field and the magnetic field show almost the same behavior; however, the temperature distribution of the mixture differs significantly between the electric and magnetic fields.

CaCO_3_, which has low electrical conductivity, can be heated only by a microwave electric field. Microwave magnetic field heating heats the mixture without heating CaCO_3_. Further, components that can be heated by microwave electric and magnetic fields in the mixture are carbides. Therefore, the high-temperature points observed by magnetic field heating are likely to be carbides, and the low temperature points are likely to be CaCO_3_ and NaCl. Thus, the phenomenon can be explained well if it is considered that CaCO_3_ does not decompose because the region near the carbide is heated and sintered in the mixture.

## Conclusions

Carbon fly ash containing CaCO_3_ with and without water was sintered using microwave irradiation. The mixture was heated from 300 to 1000 °C in approximately 100 s using microwave irradiation. The results of the resonance perturbation method indicated that the permittivity complex part of the mixture is better when a higher amount of fly ash containing a large amount of unburned carbon is added; further, CaCO_3_ and NaCl barely absorb microwaves. For the reaction behavior, a sintered body containing a large amount of aragonite was synthesized by adding water. The carbon storage content of the sintered body decreased with an increase in the heating time, and the sintered body at approximately 1 min showed the highest carbon content compared to those at other heating times. Further, the sintered body heated by the microwave magnetic field contained more CaCO_3_ than that heated via electric field heating. The results of the exhaust gas analysis showed that microwave magnetic field heating releases CO_2_ at a temperature as high as 400 °C. In addition, the two-dimensional two-color thermometer results suggested that the microwave magnetic field heating has a smaller superheat point of micrometers or less than that obtained with the microwave electric field heating. Thus, it is necessary to proceed with the analysis using mesoscale thermodynamics.

The diffusion of water can be suppressed and CaCO_3_ is formed in the temperature drop upon heating the mixture at high speed with microwaves. The microwaves selectively heat the carbides in the mixture to suppress the decomposition of CaCO_3_ and carbon combustion. This technology, which makes good use of the effects of high-speed and selective heating, is expected to be expanded to technologies for converting CaCO_3_ obtained from concrete and slag into carbon storage structural materials.

## Supplementary Information

Supplementary Information.

## Data Availability

All data generated or analyzed during this study are included in this published article (and its Supplementary Information files).
