# Supplementary material for: Manufacturing carbon storage sintered body using microwave-selective and high-speed heating techniques
Source: Sci Rep. 2023 Mar 29;13:5122. doi: 10.1038/s41598-023-32136-z (PMC10060211; doi:10.1038/s41598-023-32136-z)
Supplement: Supplementary file 1 — Supplementary Information. [file 41598_2023_32136_MOESM1_ESM.docx]

Supplementary Material

Manufacture of carbon storage sintered body using microwave-selective and high-speed heating techniques

**K. Kashimura^a^*, A. Oshita^a^, T. Miyata^a^, S. Segawab, H. Yokawa^a^, K. Tendo^b^ and K. Kurooka^b^**

(a)

(b)

Table S1: Mass ratio of each element measured by XRF heated at (a) *E_max_* and (b) *H_max_*.


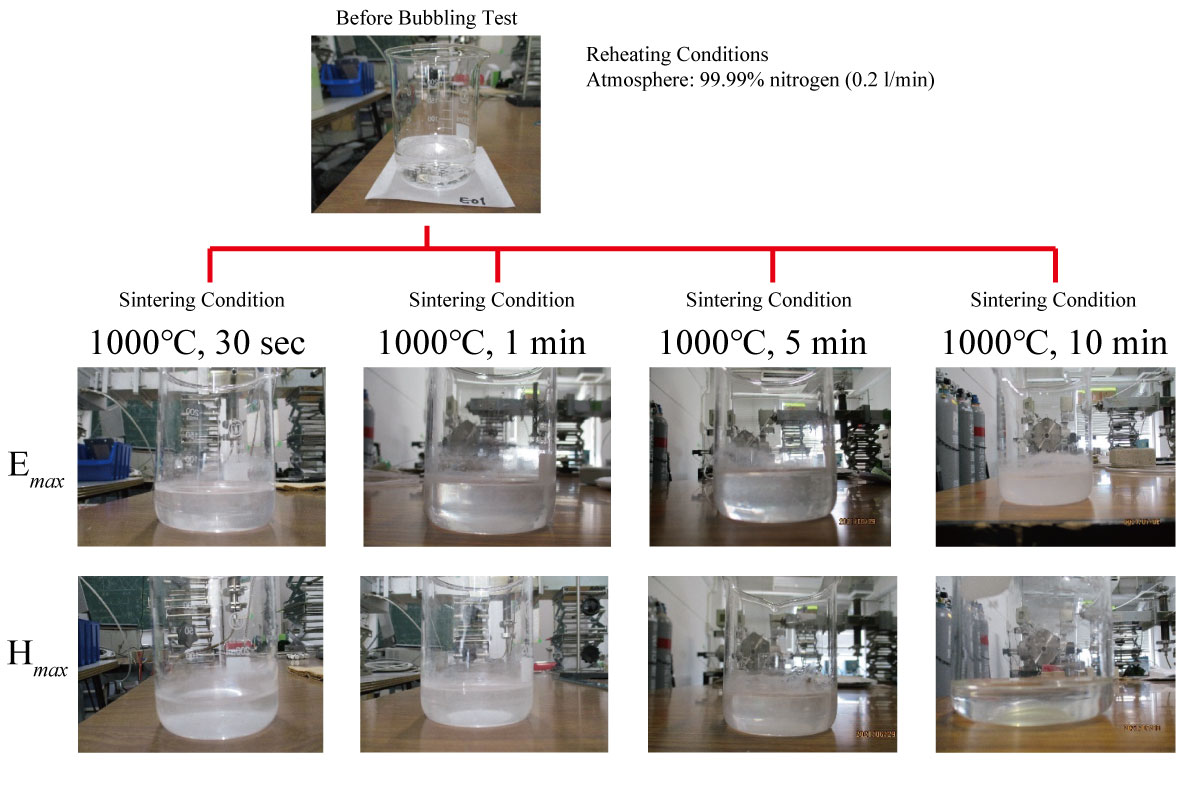


Figure S1: Exhaust gas CO_2_ confirmation test when reheating the sintered body: reheating temperature: 1000 °C, atmosphere: 4N- N_2_, flow rate: 0.2 L/min.

(a)


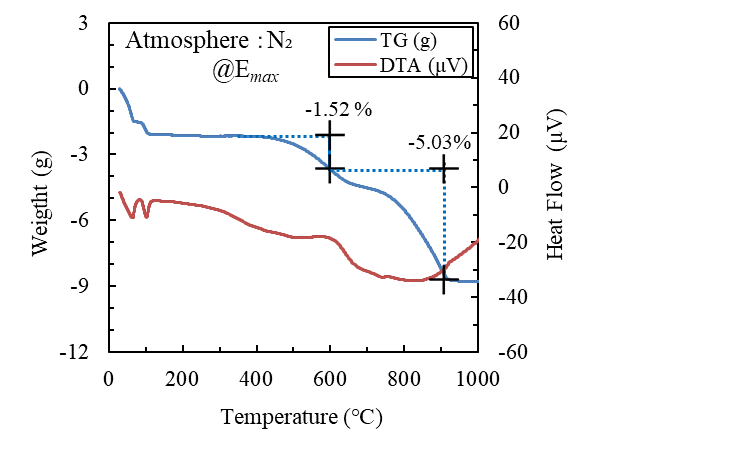


(b)


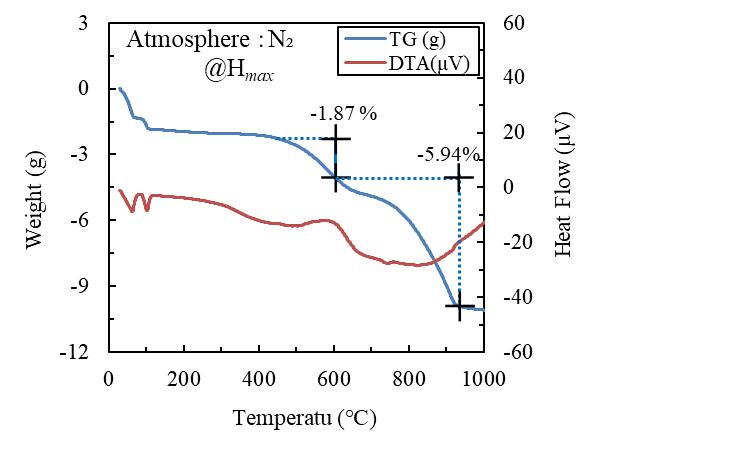


Figure S2: TG-DTA result of sintered bodies (1000 °C, 30 s). The sintered body heated by a microwave magnetic field (a) contains more CO_2_ than the sintered body heated by the microwave electric field (b).
